# Brain mapping for lower-grade glioma around Wernicke’s area

**DOI:** 10.3171/2024.10.FOCVID24101

**Published:** 2025-01-01

**Authors:** Vich Yindeedej, Kosuke Nakajo, Yuta Tanoue, Tsutomu Ichinose, Takeo Goto

**Affiliations:** 1 Department of Neurosurgery, Osaka Metropolitan University Graduate School of Medicine, Osaka, Japan; and; 2 Division of Neurosurgery, Department of Surgery, Thammasat University Hospital, Faculty of Medicine, Thammasat University, Pathumthani, Thailand

**Keywords:** brain mapping, lower-grade glioma, Wernicke’s area

## Abstract

Surgery of lesions around Wernicke’s area is challenging for several reasons. The anatomical boundaries are not clearly defined, necessitating functional identification in addition to anatomical landmarks. There are potential complications secondary to injury of the surrounding structures. This challenge is more substantial when dealing with lower-grade gliomas in this region, as surgeons must balance the extent of resection with the risk of potential injury to the patient, especially considering that there may be other treatment options for the management of residual tumors. In this video, the authors present a case illustration, discussing surgical considerations and strategy.

The video can be found here: https://stream.cadmore.media/r10.3171/2024.10.FOCVID24101

## Transcript

### 0:24 Anatomy of Wernicke’s Area.

 The anatomical boundaries of Wernicke’s area are not clearly defined in the literature. Generally, it is located in part of the inferior parietal lobule and the posterior part of the superior and middle temporal gyrus.^1^ Wernicke’s area is theoretically connected with the arcuate fasciculus.^2^ For precise mapping and safe preservation during surgery, both anatomical and functional identification are useful.

### 0:52 Function of Wernicke’s Area.

 Originally, Wernicke’s area refers specifically to a region in the dominant hemisphere involved in complex language processing, mainly comprehension. In the nondominant hemisphere, the homologous area is responsible for spatial awareness and the concept of body image.^3^ Deficits in this area on the nondominant side can cause spatial neglect syndrome.

### 1:16 Possible Complications.

Wernicke’s area is surrounded by several eloquent areas, and any damage can lead to complications. These include sensory disturbances from the sensory cortex anteriorly, symptoms of Gerstmann’s syndrome from the inferior parietal lobule,^4^ language disturbances from Wernicke’s area itself, and auditory disturbances from Heschl’s gyrus.

### 1:39 Subcortical White Matter.

Several related subcortical white matter tracts, such as the arcuate fasciculus, as a part of the dorsal stream of language function. Symptoms of aphasia could occur if the arcuate fasciculus is damaged, especially conductive aphasia. The superior longitudinal fasciculus and the sagittal stratum, which is the group of multiple white matter tracts located at the lateral wall of the atrium,^5^ are also crucial. Preserving these structures during surgery near Wernicke’s area is both critical and challenging.

### 2:12 Patient History.

We present a case of a 48-year-old female truck driver with a history of a first episode seizure. Her neurological and cognitive examinations were normal.

### 2:24 Imaging.

MRI FLAIR imaging revealed a hyperintense lesion in Wernicke’s area on the left supramarginal and angular gyrus, invading part of the superior parietal lobule. There was no T2-FLAIR mismatch or contrast enhancement on the MRI, and no calcification on CT. Based on these data, the preoperative diagnosis was astrocytoma grade 2, with a differential diagnosis of oligodendroglioma grade 2.

### 2:54 Surgical Consideration.

For surgical consideration, we must balance the extent of resection with the risk of neurological deficits due to possible injury to Wernicke’s area and surrounding aforementioned structures. The type of tumor also influences our strategy, as oligodendroglioma grade 2 responds well to chemotherapy and radiation,^6^ unlike astrocytoma, which shows only moderate response to radiation and limited evidence of chemotherapy efficacy.^7,8^ Additionally, her occupation as a truck driver requires specific functions such as an intact visual field; deficits like homonymous hemianopia or sensory disturbances could hinder her ability to work.

### 3:37 Strategy.

For these reasons, we plan a safe maximal resection under awake craniotomy, monitoring language and visual fields to prevent homonymous hemianopia, and preserving sensory function and Gerstmann’s syndrome.

### 3:51 Intraoperative Brain Mapping Task.

We plan several brain mapping tasks, including dual task, which examine picture naming and motor function for monitoring arcuate fasciculus. Additionally, repetition, word generation, and free talk are planned to monitor this tract. Any level of speech disturbance, including anomia, dysphasia, aphasia, or even dysarthria, was documented, ensuring we did not go beyond the boundary. Pyramid and palm tree test for semantic paraphasia for monitoring inferior fronto-occipital fasciculus. Four-screen test for visual field defects as the function of optic radiation. Sensory disturbance monitoring for somatosensory cortex and tracts. Finally, calculation for one-digit and two-digit results, left-right disorientation, finger agnosia, and writing to monitor Gerstmann’s syndrome.

### 4:48 Stimulation Setting.

We use bipolar stimulation to ensure precise stimulation in both cortical and subcortical mapping with an amplitude of 4 mA, a pulse duration of 0.5 msec, and a pulse rate of 50 Hz. If residual tumor was clearly present, we considered lowering the amplitude to 2 mA to facilitate more radical tumor removal.

### 5:13 Surgical Procedure.

The patient was placed in the lateral position with her head rotated approximately 90° to the contralateral side and fixed with a Mayfield head holder. Wide craniotomy and dural incision were performed at the lesion site.

Cortical mapping was conducted to ensure safe corticotomy. Incorrect calculations in this area led us to choose a different gyrus for corticotomy. After confirming negative mapping, safe tumor debulking was performed.

Free talk was useful during the operation.

Cortical mapping was repeated at the supramarginal gyrus. After confirming negative stimulation, the resection was continued safely.

Repetition was also checked.

We checked her sensation on the posterior side of the postcentral sulcus.

Due to anomia, we would not resect beyond this point.

Now we were close to the optic radiation. The tumor around here was relatively harder. She saw some abnormal light, which indicated the stimulation to the optic radiation. Therefore, this area was the limit of resection.

### 8:23 Postoperative MRI.

Postoperative MRI showed the tumor was partially removed. The remnant was found in the superior parietal lobule and the inferior side of the optic radiation. The pathological diagnosis was astrocytoma grade 2, *IDH*-mutant, with intact 1p/19q, *MGMT*-methylated, no homozygous deletion of *CDKN2A*, and no *TERT* promoter mutation.

### 8:52 Postoperative Status.

Immediately postoperative, the patient showed anomia, phonemic paraphasia, repetition disturbance, comprehension deficit, and severe Gerstmann syndrome. Two weeks after surgery, word generation was still deficit and there were minor repetition issues, her paraphasia nearly resolved. Acalculia persisted, and finger agnosia nearly resolved.

### 9:19 Follow-Up.

At the 6-month follow-up, she had right inferior quadrantanopia. The Standard Language Test of Aphasia showed deficits in word comprehension and calculation. The Revised Hasegawa’s Dementia Scale was normal, and the Mini-Mental State Examination scored 29. Her Wechsler Memory Scale and Wechsler Adult Intelligence Scale showed minimal decline compared to preoperative status.

### 9:46 Conclusion.

In conclusion, surgery for lower-grade glioma around Wernicke’s area is challenging due to potential neurological deficits, including language deficits, visual field defects, sensory disturbances, and Gerstmann’s syndrome. Awake craniotomy and brain mapping are essential for surgery in this area, as illustrated by our case. Balancing tumor resection extent with the risk of potential injury is crucial.

## Disclosures

The authors report no conflict of interest concerning the materials or methods used in this study or the findings specified in this publication.

## Author Contributions

Primary surgeon: Nakajo. Assistant surgeon: Tanoue, Ichinose. Editing and drafting the video and abstract: Nakajo, Yindeedej. Critically revising the work: Nakajo, Yindeedej, Tanoue. Reviewed submitted version of the work: Nakajo, Yindeedej, Tanoue. Approved the final version of the work on behalf of all authors: Nakajo. Supervision: Goto.

## Supplemental Information

### Patient Informed Consent

The necessary patient informed consent was obtained in this study.
